# LFA-1 (CD11a/CD18) and Mac-1 (CD11b/CD18) distinctly regulate neutrophil extravasation through hotspots I and II

**DOI:** 10.1038/s12276-019-0227-1

**Published:** 2019-04-09

**Authors:** Young-Min Hyun, Young Ho Choe, Sang A. Park, Minsoo Kim

**Affiliations:** 1 Department of Anatomy, Yonsei University College of Medicine, Seoul, Republic of Korea; 2 Brain Korea 21 Plus Project for Medical Science, Yonsei University College of Medicine, Seoul, Republic of Korea; 3 Department of Microbiology and Immunology, David H. Smith Center for Vaccine Biology and Immunology, University of Rochester, Rochester, NY USA; 4 Present Address: School of Medicine, CHA University, Seongnam, South Korea

**Keywords:** Acute inflammation, Imaging the immune system, Neutrophils

## Abstract

Precise spatiotemporal regulation of leukocyte extravasation is key for generating an efficient immune response to injury or infection. The integrins LFA-1(CD11a/CD18) and Mac-1(CD11b/CD18) play overlapping roles in neutrophil migration because they bind the same as well as different ligands in response to extracellular signaling. Using two-photon intravital imaging and transmission electron microscopy, we observed the existence of preferred sites for neutrophil entrance into the endothelial cell monolayer and exit from the basement membrane and pericyte sheath during neutrophil extravasation, namely, hotspots I and II, by elucidating distinctive roles of LFA-1 and Mac-1. To penetrate the vascular endothelium, neutrophils must first penetrate the endothelial cell layer through hotspot I (i.e., the point of entry into the endothelium). Neutrophils frequently remain in the space between the endothelial cell layer and the basement membrane for a prolonged period (>20 min). Subsequently, neutrophils penetrate the basement membrane and pericyte sheath at hotspot II, which is the final stage of exiting the vascular endothelium. To further investigate the roles of LFA-1 and Mac-1, we newly generated LFA-1 FRET (CD11a-YFP/CD18-CFP) mice and Mac-1 FRET (CD11b-YFP/CD18-CFP) mice. Using both FRET mice, we were able to determine that LFA-1 and Mac-1 distinctly regulate the neutrophil extravasation cascade. Our data suggest that the vascular endothelium functions as a double-layered barrier in the steps of neutrophil extravasation. We propose that the harmonized regulation of neutrophil penetration through the endothelium via hotspots I and II may be critical for vascular homeostasis during inflammation.

## Immune response: White blood cells follow the leader

In response to infection or tissue damage, white blood cells known as leukocytes exit blood vessels at specific sites, “hotspots.” Many leukocytes follow each other out through the same hotspot. As part of the body’s initial response to infection or tissue damage, leukocytes are carried to infected or damaged tissues by blood vessels. To fight infection, they must exit the vessels, but how they exited was poorly understood. Young-Min Hyun at the Yonsei University College of Medicine in Seoul, South Korea and co-workers used advanced imaging techniques to visualize leukocyte delivery. They found that many leukocytes use a single hotspot to enter the blood vessel wall, travel through the wall’s interior, and then exit the wall at another hotspot. Using hotspots is thought to minimize the number of perforations in the vessel wall, maintaining vessel integrity. These results illuminate a key aspect of how efficiently the body fights infection from the viewpoint of leukocyte migration.

## Introduction

The leukocyte adhesion cascade inside blood vessels includes rolling, adhesion, and crawling^1–4^. The subsequent stage is transendothelial migration via the paracellular pathway^5^ or the transcellular pathway^6,7^. Upon completion of transendothelial migration, leukocytes encounter the endothelial basement membrane and pericyte sheath. Pericytes and other perivascular resident cells are actively involved in leukocyte migration^8^. Penetrating through the endothelial cell layer is a rapid stage of leukocyte migration in the migration cascade through the vascular endothelium, but penetration of the endothelial basement membrane takes much longer^9^. In contrast to the well-studied early stages of leukocyte migration, the mechanisms underlying the termination of transendothelial migration and subsequent migration through the basement membrane remain elusive. That gap is due to the infeasibility of generating physiologically relevant basement membrane for in vitro experiments^10^. Using two-photon intravital imaging, we previously revealed that, after the completion of transendothelial migration and prior to proceeding toward the interstitial area, leukocytes detach their trailing edge from the basolateral side of the endothelial layer and/or basement membrane^9^. Various signaling pathways and adhesion molecules are involved in these steps^11^. Thus leukocyte infiltration involves complicated sequential steps that should be well orchestrated for a proper immune response to injury or infection. Therefore, intravital imaging is a useful experimental tool for observing the actual phenomena of leukocyte extravasation.

Among leukocyte subsets, neutrophils are the first major innate immune cell population that extravasates from the vessel, through the vascular endothelium, and finally to the inflamed interstitium during the inflammatory response. For an efficient immune response to be initiated by neutrophil extravasation, spatiotemporal regulation of neutrophil migration is critical for host defenses. LFA-1 (CD11a/CD18) and Mac-1 (CD11b/CD18) play distinct roles at each stage of the neutrophil extravasation cascade but share CD18 as their heterodimeric component, which may allow them to have similar functions^12–14^. Thus the functional study of the distinct roles of LFA-1 and Mac-1 in each step of the neutrophil extravasation cascade is important for understanding the migratory strategy of neutrophils during the innate immune response. Previous studies have reported that neutrophils penetrate through the gaps between pericytes, which are aligned with the vascular basement membrane or the lower deposition of certain basement membrane constituents^15–17^. Proebstl et al. introduced the hotspot concept for the infiltration of multiple neutrophils through the same pericyte gap in a sequential manner^18^. They also showed that abluminal crawling patterns of migrating neutrophils, such as directionality and speed, are affected by pericyte-expressed intercellular adhesion molecule-1 and its receptors Mac-1 and LFA-1 in response to tumor necrosis factor (TNF) and interleukin (IL)-1β^18^.

In this study, using two-photon intravital microscopy in live mice, we expanded the hotspot concept to a preferred route through hotspot I as an entrance and hotspot II as an exit for neutrophil extravasation. Based on the data from the current study, we propose that the vascular endothelium is a double-layered barrier through which neutrophils have to pass during extravasation. After transendothelial migration at hotspot I, neutrophils remain in the endothelial basement membrane for a period of time. Based on two-photon intravital imaging and transmission electron microscopy, we discovered another preferred site, hotspot II, at which neutrophils migrate to overcome the endothelial basement membrane and pericyte sheath barrier. Several neutrophils continuously penetrate the endothelial basement membrane at the same site. Thus neutrophils rely on the penetration hotspots at the early stage of inflammation. The use of preferred sites for extravasation likely minimizes the disruption of the vascular wall, resulting in maintenance of vascular barrier function. Thus we suggest that these hotspots may be important for vascular homeostasis and maintaining vascular barrier function.

Thereafter, we further investigated how LFA-1 and Mac-1 regulate neutrophil extravasation in a time-lapse manner, specifically focusing on transendothelial migration, perivascular crawling, and uropod elongation. LFA-1 and Mac-1 are key integrins that regulate leukocyte adhesion and migration in inflamed tissues. Despite considerable advances in our understanding of leukocyte migration, the real-time regulation of the leukocyte integrins LFA-1 and Mac-1 in vivo is not well characterized. From the technical perspective, fluorescence resonance energy transfer (FRET) is the only method that may be used to study integrin activation in live cells in real time^19–23^. Since functional studies of integrin using FRET have been applied in vitro, we designed integrin FRET sensors based on the integrin activation model to detect the conformational state of LFA-1 and Mac-1 in the immune cells of live animals by reporting the relative distance between the cytoplasmic tails of the α and β subunits. By using two-photon intravital ratiometric analysis of cyan fluorescent protein/yellow fluorescent protein (CFP/YFP) in fluorescent neutrophils from LFA-1 FRET (CD11a-YFP/CD18-CFP) mice and Mac-1 FRET (CD11a-YFP/CD18-CFP) mice, we successfully determined that the high-affinity LFA-1 and clustered Mac-1 are distinctly regulated throughout the neutrophil extravasation cascade and during the entrance into and exit from the double-layered endothelial barrier.

## Materials and methods

### Animals

C57BL/6 and LysM-GFP^24^ were purchased from Jackson Laboratory and maintained in a specific pathogen-free environment at the University of Rochester’s Animal Facility. CD11b-mYFP knock-in mice were generated at the Gene Targeting and Transgenic Core Facility at the University of Rochester using gene targeting techniques similar to those previously used in our laboratory to generate the CD18-mCFP and CD11a-mYFP knock-in mice and the K562 Mac-1 FRET cell line^9,21,25^. All mice were backcrossed to C57BL/6 for at least six generations. LFA-1 FRET (CD11a-mYFP/CD18-mCFP) mice were subsequently generated by mating the CD11a-mYFP and CD18-mYFP knock-in mice. Mac-1 FRET (CD11b-mYFP/CD18-mCFP) mice were generated by mating the CD11b-mYFP and CD18-mCFP knock-in mice.

### Two-photon intravital microscopy of blood vessels in the mouse cremaster muscle

To visualize neutrophil motility during extravasation, two-photon intravital microscopy was performed using an Olympus FV1000-AOM multi-photon system equipped with a ×25 NA 1.05 water immersion objective. For two-photon excitation, a Spectra-Physics MaiTai HP Ti:Sa Deep See laser system was tuned to 900 nm for green fluorescent protein (GFP) and Texas Red and 840 nm for CFP, YFP, and Texas Red. The images were acquired at a resolution of 256 × 256 pixels with a pixel dwell time of 2 μs using step sizes of 1 μm to a depth of 25–30 μm every 30 s. To image the cremaster muscle, the mice were initially anesthetized via intraperitoneal injection of pentobarbital sodium (a dose of 65 mg/kg), and the hair on the skin of the imaging area was removed. To image the cremaster blood vessels, the right cremaster muscle was exteriorized and covered with pre-warmed physiological solution at 36 °C. The solution contained the following (in mM): NaCl, 131.9; KCl, 4.7; CaCl_2_, 2.0; MgSO_4_, 1.2; and NaHCO_3_, 18 (pH 7.4). The solution was equilibrated with gas containing 0% O_2_, 5% CO_2_, and 95% N_2_, to maintain the tissue PO_2_ <15 Torr. The mice were subsequently placed on a custom platform, and anesthesia was maintained with isoflurane for restraint and to avoid inflicting psychological stress and pain on the animal during imaging. The core body temperature of the mice was maintained using a warming pad set to 37 °C. Texas Red/dextran (70,000 Mw; Invitrogen) was injected intravenously (20 mg/kg) via the femoral vein to label the blood vessels immediately prior to imaging. The blood vessels were stimulated by superfusion of chemokines (1 nM C-X-C chemokine motif ligand 2 (CXCL2)) or a bacterial chemoattractant (1 μM *N*-formylmethionyl-leucyl-phenylalanine (fMLP)). For TNFα stimulation, TNFα (0.5 μg in 250 μL of saline) was intrascrotally injected 4 h prior to in vivo imaging. When the imaging experiment was finished, the mice were euthanized by CO_2_ inhalation.

### Two-photon intravital ratiometric analysis of CFP/YFP in neutrophils of LFA-1 FRET and Mac-1 FRET mice

Two-photon intravital microscopy was performed to image CFP and YFP in neutrophils of LFA-1 FRET and Mac-1 FRET mice stimulated with fMLP, as described above. Ratiometric analysis of CFP/YFP was performed to reflect the conformation status of LFA-1 and Mac-1.

### Electron microscopy

Leukocyte extravasation was first observed in the fMLP-stimulated cremaster venules of C57BL/6 mice using two-photon intravital microscopy. The cremaster muscle was immediately dissected from the body post-euthanasia and fixed with 2.5% glutaraldehyde. The tissue was further processed for transmission electron microscopy in the Electron Microscope Research Core at the University of Rochester.

### Imaging data analysis

Fiji/ImageJ software was used for image analysis, and the basic image processing Volocity software (PerkinElmer, USA) was used for imaging data analysis.

### Mouse neutrophil preparation

Mouse neutrophils were isolated from bone marrow^26^. Briefly, the femurs and tibias were harvested and stripped of all muscle and sinew. The bone marrow was then flushed out with 10 mL of RPMI medium containing 5% fetal bovine serum (Invitrogen, Carlsbad, CA, USA) on ice. The cells were pelleted by centrifugation for 3 min at 1500 rpm, and the erythrocytes were depleted. Neutrophils were then prepared from mouse bone marrow using the EasySep Neutrophil Enrichment Kit (Stemcell Technologies).

###  Western blotting

Neutrophils were isolated from the bone marrow of C57BL/6, LFA-1 FRET, and Mac-1 FRET mice. Western blotting was used to verify that the expression of CD11a-mYFP, CD11b-mYFP, and CD18-mCFP in knock-in mouse neutrophils was normal compared with WT neutrophils^27^.

### Statistical analysis

Graphical data generation and basic statistical analyses were performed using the GraphPad Prism 6 software. Comparisons between separate groups were conducted using Student’s *t* test or Wilcoxon rank-sum test for continuous variables and chi-square test or Fisher’s exact test for categorical variables. *P* values <0.05 were considered to indicate statistically significant differences.

## Results

### Neutrophils penetrate the endothelium at hotspots during the early stage of inflammation

**Fig. 1:**
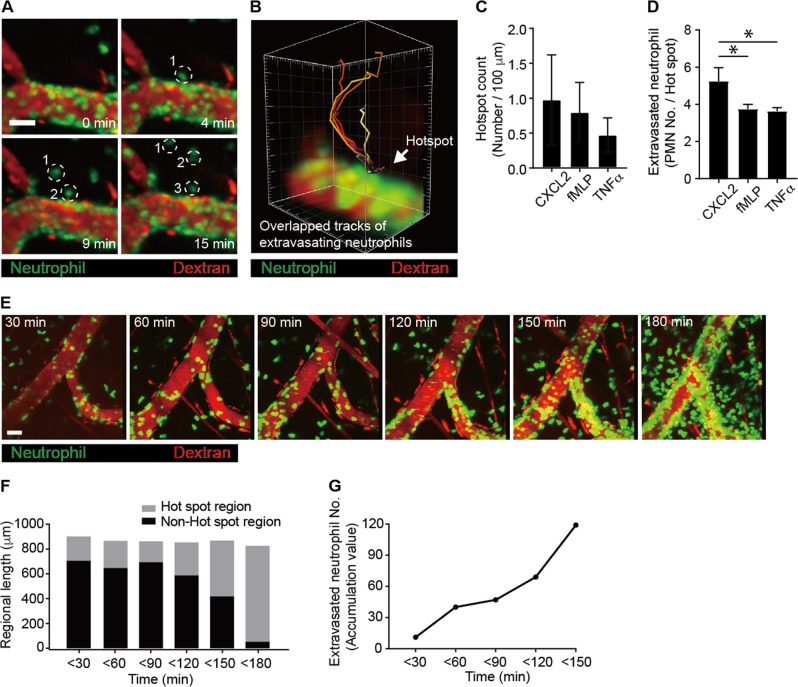
Neutrophils penetrate the endothelium at hotspots during the early stage of inflammation. **a** Analysis of neutrophil infiltration at a hotspot from the cremaster muscle blood vessel stimulated with *N*-formylmethionyl-leucyl-phenylalanine (fMLP). Dotted circles indicate neutrophils from the same hotspot. Scale bar, 50 μm. **b** The tracks of six extravasating neutrophils overlapped, indicating that neutrophils penetrated through a hotspot. **a**, **b** were analyzed from Video 1. **c** Hotspots were counted per 100 μm of blood vessels upon stimulation with C-X-C chemokine motif ligand 2 (CXCL2), fMLP, and tumor necrosis factor α (TNFα). **d** The numbers of neutrophils undergoing extravasation per hotspot were determined upon stimulation with CXCL2, fMLP, and TNFα. *P < 0.005. **e** Neutrophils penetrated the endothelial cell layer at a hotspot in the early stage of inflammation. Two-photon intravital imaging of LysM-GFP mice was performed upon stimulation with fMLP. Scale bar, 30 μm. See Video 2. **f** Analysis of neutrophil infiltration at a hotspot region vs. non-hotspot region at each time point upon stimulation with fMLP. **g** The accumulated number of neutrophils undergoing extravasation with increasing time after stimulation. **f**, **g** were analyzed from Video 2. Representative data from five independently repeated experiments are shown

Following crawling along the blood vessels during inflammation, neutrophils adhere to the luminal surface of the blood vessels and subsequently undergo transendothelial migration via the paracellular pathway^5^ or the transcellular pathway^6,7^. During the extravasation cascade through the inflamed vessels, intravascularly crawling neutrophils need to find a site for transendothelial migration regardless of the paracellular pathway or the transcellular pathway. Using two-photon intravital imaging of neutrophil extravasation in the stimulated blood vessels, we defined a specific site on a blood vessel as a hotspot when at least three continuous neutrophils infiltrated at that same site (Fig. 1a and Video 1). The three-dimensional image accumulated in a time-lapse manner over 30 min further confirmed the hotspot, through which six neutrophils continuously penetrated the endothelium (Fig. 1b and Video 1). Although the hotspots were not uniformly distributed in the inflamed vessel, one or two hotspots were usually found per 100 μm of inflamed venules (Fig. 1c). When we tested different stimuli (i.e., chemokine [CXCL2], cytokine [TNFα], and bacterial peptide [fMLP]), the preferred site for the neutrophil extravasation hotspot was generally observed (Fig. 1d). We further found that neutrophils penetrating the endothelium frequently used the same site previously used by other neutrophils for transendothelial migration (Fig. 1e–g and Video 2). These selective migratory sites for neutrophils have been termed “hotspots”^18^. Interestingly, we found that, for the first 120 min after stimulation of the blood vessel, only a small portion of the analyzed blood vessel length was used as hotspots that allowed the extravasation of most neutrophils (Fig. 1e). Occasionally, as more neutrophils underwent transendothelial migration, adjacent hotspots merged with each other and consequently became wider (<150 min). When the vessels ultimately became severely inflamed, which usually occurred 120–240 min after stimulation, they were almost completely covered with firmly adhering as well as extravasating neutrophils (Fig. 1e, f). This result implies that the hotspots play a meaningful role as the preferred sites of neutrophil infiltration through the endothelium only during the early stage of inflammation (or during mild inflammation). The hotspots lost their importance as the preferred exit sites when the acute inflammation fully progressed, as neutrophil infiltration then occurred over a much wider blood vessel area; eventually, neutrophils adhered to most regions of the blood vessels. In summary, the hotspots were the initial sites for selective extravasation of neutrophils in the early stage of inflammation and ceased to be the preferred sites for neutrophil extravasation in severely inflamed vessels.

### Vascular endothelium is a double-layered barrier for neutrophil extravasation

Sequential steps of neutrophil extravasation have been intensively studied, with a focus on the interactions of adhesion molecules with ligands, chemokine signaling, and the role of perivascular macrophages^28,29^. In particular, using both two-photon microscopy and electron microscopy, research has indicated that endothelial domes are formed during neutrophil transendothelial migration in mice^30^. A study using electron microscopy also showed that human neutrophils completed transcellular migration through individual endothelial cells^31^. In addition to these studies on transendothelial migration of neutrophils, we further performed both two-photon microscopy and electron microscopy to investigate how neutrophils are processed, step by step, for spatiotemporal extravasation through their distinct layers in the endothelium (e.g., the endothelial cell layer and the basement membrane).

**Fig. 2:**
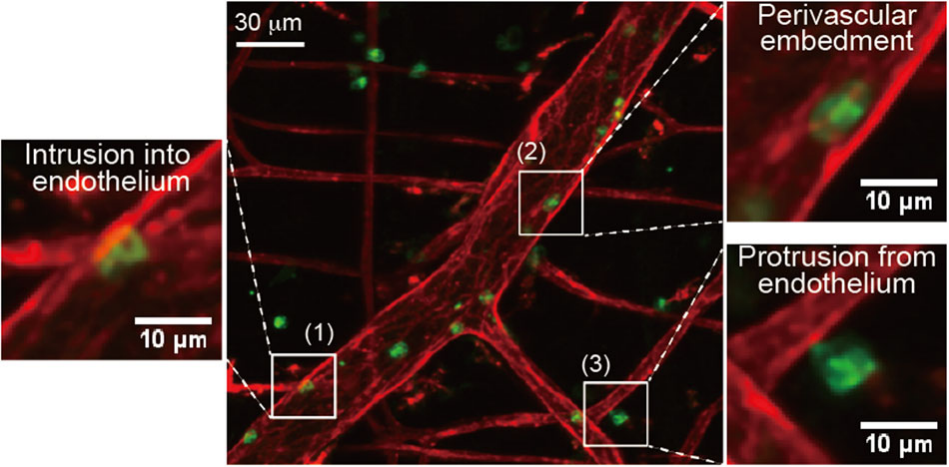
Visualization of the substeps of neutrophil extravasation by two-photon microscopy. Analysis of neutrophil infiltration visualized from three-dimensional two-photon images of anti-CD31 antibody-labeled blood vessels in mouse cremaster upon stimulation with *N*-formylmethionyl-leucyl-phenylalanine: (1) intrusion into the endothelium, in which the neutrophil cell body started to penetrate the endothelial cell layer; (2) most of the neutrophil cell body then was colocalized within the CD31-labeled endothelial cell layer; (3) as the final step, the tailing edge of the extravasating neutrophil was attached. Please see Video 3

First, we confirmed that three-dimensional two-photon images of CD31-labeled blood vessels in LysM-GFP mouse cremaster visualized each step of neutrophil extravasation. As sequential steps of neutrophil extravasation, we could observe intrusion into the endothelium, in which neutrophils started to penetrate into the endothelial cell layer (Fig. 2 (1) and Video 3). Most of the neutrophil cell body was then colocalized within the CD31-labeled endothelial cell layer (Fig. 2 (2) and Video 3). In the final step, we also observed attachment of the tailing edge of the extravasating neutrophils (Fig. 2 (3) and Video 3). These data are consistent with previous results on the entrance of neutrophils into the endothelial cell layer^30^.

**Fig. 3:**
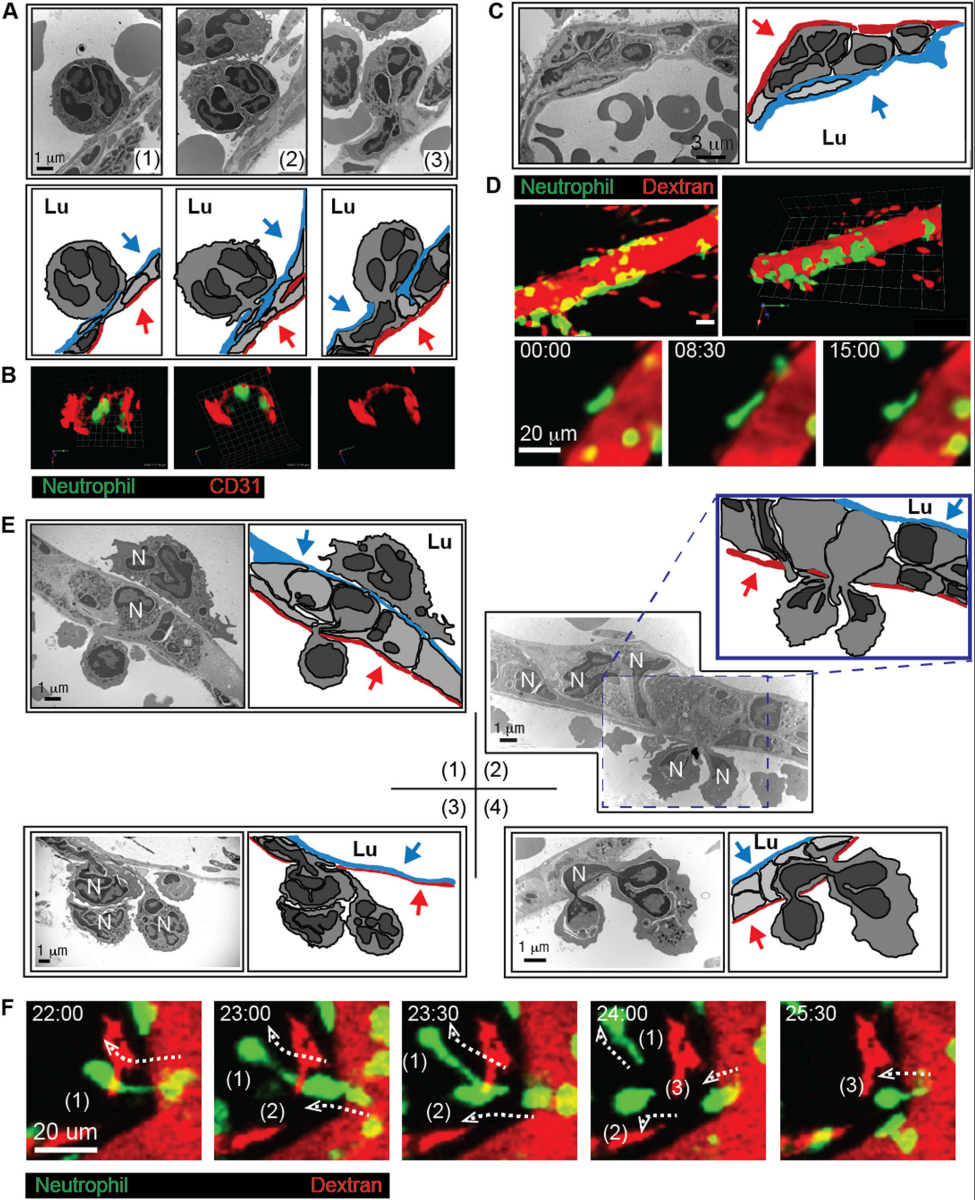
The vascular endothelium is a double-layered barrier for neutrophil extravasation. **a** Transmission electron microscopy revealed a gradual intrusion of neutrophils into the endothelial cell layer (upper panel). The cartoons of the transmission electron micrographs from the upper panel indicate the endothelial cell layer in red and pericytes in blue (lower panel). **b** Two-photon three-dimensional microscopy revealed the presence of transendothelial migratory neutrophils. See Video 4. **c** Transmission electron microscopy revealed neutrophil embedment in the endothelial basement between the endothelial cell layer and pericytes (upper panel). The cartoons of the transmission electron micrographs from the upper panel indicate the endothelial cell layer shown in red and pericytes in blue (lower panel). **d** Two-photon intravital microscopy revealed that neutrophils were embedded in the endothelial basement membrane. See Videos 5 and 6. **e** Transmission electron microscopy revealed that neutrophils penetrated the pericyte sheath. (1) The leading edge of an embodied neutrophil protruded from the endothelial basement membrane between the endothelial cell layer and the pericyte sheath. (2) Two neutrophils were undergoing penetration at the same site of the pericyte sheath in the panel. (3) Three neutrophils were undergoing penetration at the same site of the pericyte sheath in the panel. (4) The head and tail regions of a neutrophil are localized outside the pericyte sheath in the panel. In all of the corresponding cartoons, the endothelial cells are shown in blue, and the pericytes are in red. Blue and red arrows indicate endothelial cells and pericytes, respectively. N neutrophil, Lu lumen. **f** Two-photon intravital microscopy revealed three neutrophils that were continually penetrating the vascular endothelium at the same spot within 5 min of each other. The dotted arrows indicate the trajectories of the extravasating neutrophils. See Video 7. For all experiments, the data are representative of five independently repeated experiments with *N*-formylmethionyl-leucyl-phenylalanine stimulation

To further confirm these sequential steps during neutrophil extravasation in detail, we used electron microscopy to visualize each stage of the neutrophil extravasation cascade. Once a neutrophil firmly adhered to the luminal surface of the blood vessel, it formed a type of hook through an entrance site into the endothelial cell layer (Fig. 3a (1)). Through the entrance of the vascular endothelial cell layer, a larger portion of the neutrophil cell body became located beneath the endothelial cell layer (Fig. 3a (2, 3)). We termed the neutrophil entrance site into the endothelial cell layer hotspot I; this site represents a novel concept related to the entrance of neutrophils into the endothelial cell layer. Two-photon microscopy analysis of LysM-GFP mouse cremaster blood vessel stained with anti-CD31 antibody further confirmed neutrophil intrusion into the endothelial cell layer (Fig. 3b and Video 4). After subsequent penetration of the endothelial cell layer, many neutrophils were embedded between the endothelial cell layer and the basement membrane within the pericyte sheath (Fig. 3c). Using two-photon intravital imaging, we also confirmed the embedment of many neutrophils in the endothelial basement membrane and engagement of neutrophils in perivascular crawling for over 20 min (Fig. 3d and Videos 5, 6). Finally, neutrophils passed through the endothelial basement membrane and pericyte sheath using a specific exit site, which we termed hotspot II (Fig. 3e, f). Using electron microscopy of the mouse cremaster blood vessel, we observed the leading edge of an embodied neutrophil that protruded from the endothelial basement membrane between the endothelial cells and the pericytes (Fig. 3e (1)). Specifically, electron microscopy further revealed simultaneous penetration of two neutrophils at hotspot II, which confirmed again that hotspot II is the preferred exit site (Fig. 3e (2)). Three neutrophils leaving the endothelial basement membrane and pericyte sheath, possibly through the same site, were also detected by electron microscopy (Fig. 3e (3)). Interestingly, we observed that the two opposite edges of one neutrophil cell were located outside the pericyte sheath, with its middle portion still in the endothelial basement membrane area (Fig. 3e (4)). This phenomenon suggests that some signaling events and/or a physical barrier of the pericyte sheath induced neutrophil migration at hotspot II in the vascular barrier environment. This may have resulted from signaling and physical hindrance of the endothelial basement membrane, the pericytes, and other perivascular resident cells, such as macrophages. Using two-photon intravital imaging, we also confirmed that three neutrophils left the endothelium via the same hotspot II within <5 min, once the first neutrophil formed an elongation (for over 20 min) and left (Fig. 3f and Video 7). This phenomenon demonstrates that the pericyte sheath may also act as a physical barrier in the vascular endothelial structure and induce a signaling mechanism for the motility of neutrophils. These observations are consistent with previous reports showing that neutrophil infiltration is regulated by the spatiotemporal expression of various cytokines and chemokines by endothelial cells, pericytes, and resident macrophages^18,28,29,32^. In summary, the vascular endothelium acts as a double-layered barrier during neutrophil extravasation, and we have introduced a novel concept of the preferred site for neutrophil extravasation: hotspot I as the entrance site into the endothelial cell monolayer and hotspot II as the exit site from the endothelial basement membrane and pericyte sheath.

### Generation of CFP/YFP-conjugated LFA-1 FRET and Mac-1 FRET mice

LFA-1 (CD11a/CD18) and Mac-1 (CD11b/CD18) are key integrins that regulate leukocyte adhesion and migration in inflamed tissues. Despite considerable advances in our understanding of leukocyte migration, the real-time regulation of leukocyte integrins in vivo is not well characterized. Two-photon intravital microscopy can be combined with FRET to visualize the real-time activity of target molecules^33,34^. To take advantage of ratiometric FRET for a real-time functional study of LFA-1 and Mac-1, we designed integrin FRET sensors based on the integrin activation model, which detects the conformational state of LFA-1 and Mac-1 in the immune cells of live animals by reporting the relative distance between the cytoplasmic tails of the α and β subunits (Fig. S1A, B). Based on our previous studies of human LFA-1 and Mac-1 with FRET sensors^22,23^, we generated knock-in mice in which CD11a or CD11b were fused with mYFP and CD18 was fused with mCFP (i.e., CD11a-mYFP, CD11b-mYFP, and CD18-mCFP mice) (Fig. S1C). We previously reported that CD18-mCFP and CD11a-mYFP mice are useful for the visualization of CD18 and CD11a integrins, respectively, during leukocyte migration^9,25^. We then generated CD11b-mYFP mice in the same manner as for CD18-mCFP and CD11a-mYFP mice. We crossed these mice to generate LFA-1 FRET (CD11a-mYFP/CD18-mCFP) mice and Mac-1 FRET (CD11b-mYFP/CD18-mCFP) mice (Fig. S1C). All mice were backcrossed for at least six generations to a pure genetic background of C57B/L mice. These mice did not show any physiological abnormalities and were useful for the visualization of the subcellular distribution of LFA-1 (CD11a/CD18) and Mac-1 (CD11b/CD18) in isolated neutrophils (Fig. S1D). Western blot analysis of CFP and YFP as well as CD18 (β subunit) from these LFA-1 FRET and Mac-1 FRET mice revealed an appropriate shift of molecular masses of the α and β subunits of LFA-1 and Mac-1 associated with the size change due to CFP and YFP (Fig. S1E). We then carefully compared the intensities of CFP and YFP in neutrophils from LFA-1 FRET and Mac-1 FRET mice at various excitation wavelengths by two-photon microscopy. In the absence of stimulation, in neutrophils isolated from LFA-1 and Mac-1 FRET mice, equivalent CFP and YFP intensities were detected at 840 nm (Fig. S2). Therefore, we performed two-photon intravital imaging to calculate the ratiometric FRET (CFP/YFP) at that wavelength.

### High-affinity LFA-1 and Mac-1 clustering distinctly localize on neutrophils during transendothelial migration upon stimulation with fMLP

**Fig. 4:**
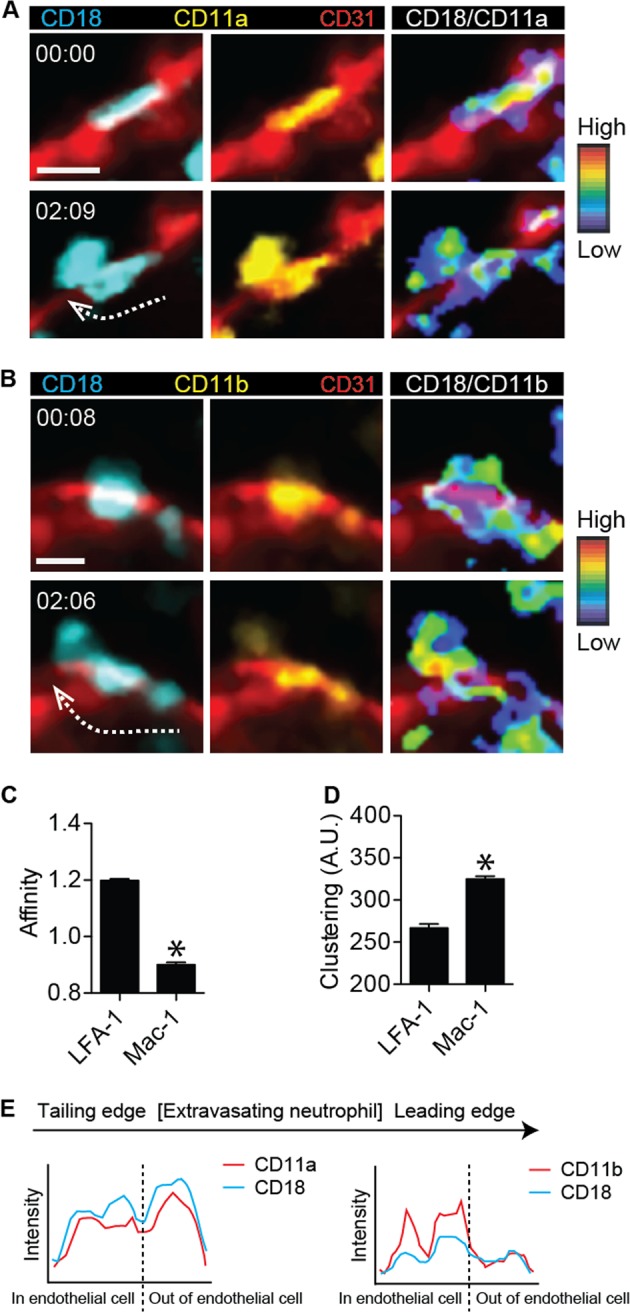
High-affinity LFA-1 and Mac-1 clustering were distinctly localized on neutrophils during transendothelial migration upon stimulation with *N*-formylmethionyl-leucyl-phenylalanine. **a** CD18 expression (left panel), CD11a expression (middle panel), and the CD18-to-CD11a ratio (right panel) were visualized at two different time points during transendothelial migration of a neutrophil in LFA-1 FRET (CD11a-mYFP/CD18-mCFP) mice. Red, endothelial cellular border imaged with an Alexa 594-stained anti-CD31 antibody. Two-photon intravital two-dimensional imaging of LFA-1 FRET mice was performed. The cyan fluorescent protein/yellow fluorescent protein (CFP/YFP) ratio is visualized using the rainbow scale. The dotted arrow shows the direction of transendothelial migration of the neutrophil. Scale bar, 10 μm. See Video 8. The images are representative of at least five independent videos. **b** CD18 expression (left panel), CD11b expression (middle panel), and the CD18-to-CD11b ratio (right panel) were visualized at two different time points during transendothelial migration of a neutrophil in Mac-1 FRET (CD11b-mYFP/CD18-mCFP) mice. Red, endothelial cellular border imaged with an Alexa-594 stained anti-CD31 antibody. Two-photon intravital two-dimensional imaging of Mac-1 FRET mice was performed. The CFP/YFP ratio is visualized using the rainbow scale. The dotted arrow shows the direction of transendothelial migration of the neutrophil. Scale bar, 10 μm. See Video 9. The images are representative of at least five independent videos. **c**, **d** The affinity and clustering of LFA-1 and Mac-1 were quantified based on CFP, YFP, and the CFP-to-YFP ratio during two-photon intravital imaging of both LFA-1 FRET and Mac-1 FRET mice. *P < 0.0001. A.U. arbitrary units. **e** The intensities of CD18 and CD11a in LFA-1 mice and CD18 and CD11b in Mac-1 FRET mice were, respectively, quantified along a line from the trailing edge to the leading edge of the transendothelial migratory neutrophils. The images are representative of analyses of at least five independent neutrophils

Using two-photon intravital ratiometric imaging of fMLP-stimulated LFA-1 FRET and Mac-1 FRET mice in conjunction with blood vessel staining by an Alexa 594-labeled anti-CD31 antibody, we successfully visualized the intensity of the α and β subunits of LFA-1 and Mac-1 and ultimately quantified the affinity and clustering of these integrins at the subcellular level (Fig. 4a, b and Videos 8, 9). Subcellular analyses of the fluorescence intensity of CFP and YFP in the generated mice revealed that LFA-1 adapted a high-affinity conformation during transendothelial migration, whereas the conformation of Mac-1 was at the basal level (Fig. 4c, d). While LFA-1 exhibited higher affinity according to the ratiometric analysis of CFP and YFP, the number of clustering Mac-1 molecules was greater than the number of clustering LFA-1 molecules based on the YFP intensity (Fig. 4c, d). Therefore, both the high affinity of LFA-1 and the clustering of Mac-1 may play critical roles in transendothelial migratory neutrophils. Ratiometric quantification of the CFP and YFP signals in neutrophils during transendothelial migration revealed high expression of YFP in the neutrophil body located in the endothelial cell layer in Mac-1 FRET mice; on the other hand, the expression of YFP in the leading edge of neutrophils located outside the endothelial cell layer was significantly decreased in Mac-1 FRET mice (Fig. 4e). However, the intensity of the YFP signal in LFA-1 FRET mice was uniformly high in the neutrophil cell body regardless of its location inside or outside the endothelial cell layer. The clustering of LFA-1 and Mac-1 revealed by YFP expression in Mac-1 FRET and LFA-1 FRET mice, respectively, suggests that LFA-1 clustering might be more important during the initial transendothelial migration than Mac-1 clustering, even at the adhesion stage outside the endothelial cell layer.

### High-affinity LFA-1 is localized at the trailing edge of neutrophils during the elongation step of extravasation upon stimulation with fMLP

**Fig. 5:**
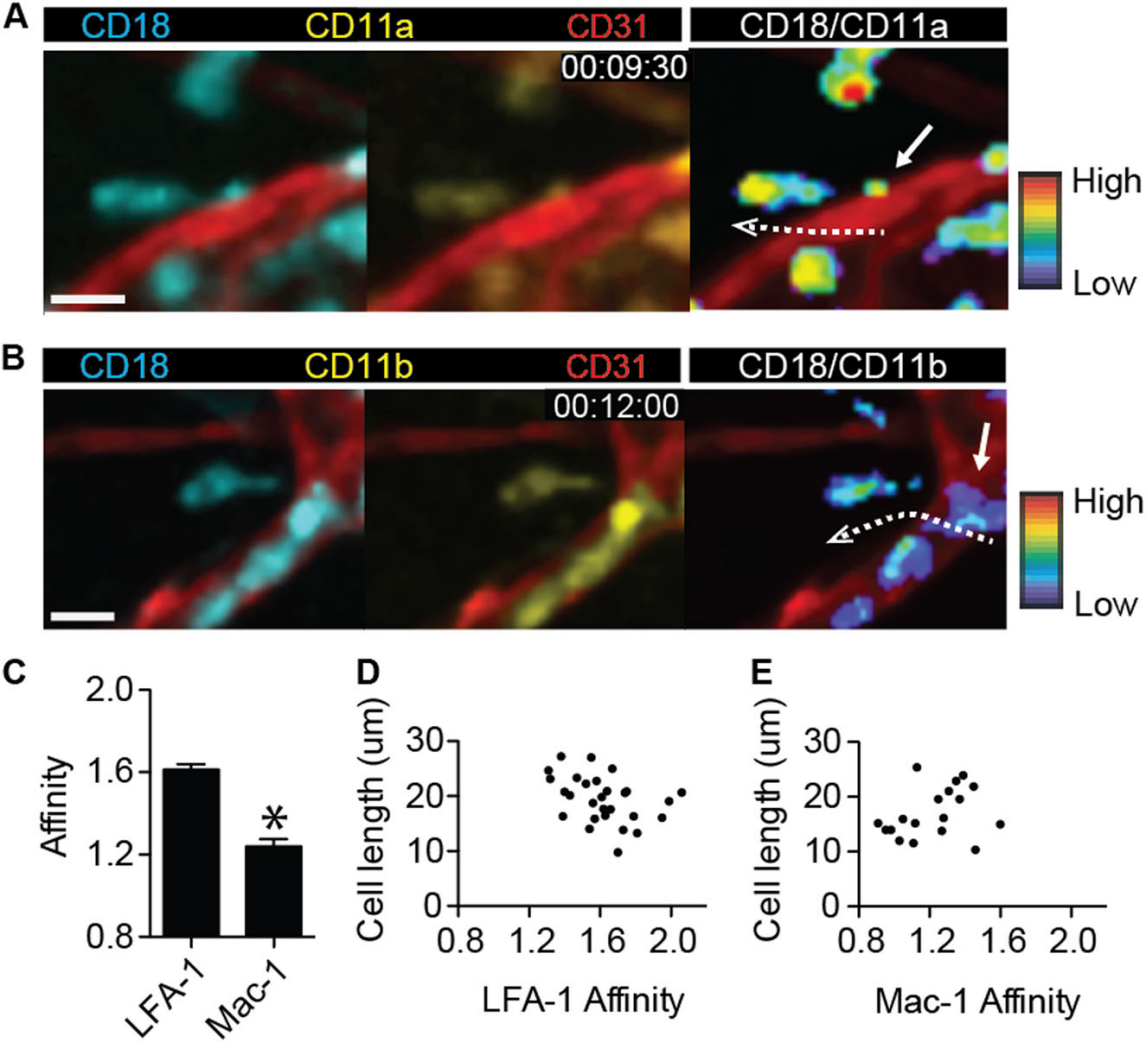
High-affinity LFA-1 is localized at the trailing edge of the neutrophil during the elongation step of extravasation upon stimulation with *N*-formylmethionyl-leucyl-phenylalanine. **a** CD18 expression (upper left panel), CD11a expression (upper right panel), Alexa 594-anti-CD31 antibody staining of an endothelial cellular border, and the CD18-to-CD11a ratio (lower right panel), visualized in the step of uropod elongation of a neutrophil in LFA-1 FRET (CD11a-mYFP/CD18-mCFP) mice. Two-photon intravital imaging of LFA-1 FRET mice was performed. The cyan fluorescent protein/yellow fluorescent protein (CFP/YFP) ratio is visualized using the rainbow scale. The arrow indicates LFA-1 affinity status at the trailing edge of an elongated neutrophil. The dotted arrow indicates the elongation of an extravasating neutrophil. Scale bar, 10 μm. See Video 10. The images are representative of at least five independent videos. **b** CD18 expression (upper left panel), CD11b expression (upper right panel), Alexa 594-anti-CD31 antibody staining of an endothelial cellular border, and the CD18-to-CD11a ratio (lower right panel), visualized in the step of uropod elongation of a neutrophil in Mac-1 FRET (CD11b-mYFP/CD18-mCFP) mice. Two-photon intravital imaging of Mac-1 FRET mice was performed. The CFP/YFP ratio was visualized using the rainbow scale. The arrow indicates the Mac-1 affinity status at the trailing edge of an elongated neutrophil. The dotted arrow indicates the elongation of an extravasating neutrophil. Scale bar, 10 μm. See Video 11. The images are representative of at least five independent videos. **c** The affinities of LFA-1 and Mac-1 were compared at the trailing edges of neutrophils during uropod elongation. *P < 0.0001. **d** Plot of the correlation of cell length with LFA-1 affinity. Data from two-photon intravital imaging of neutrophils in LFA-1 FRET mice during uropod elongation, as the final step of neutrophil extravasation, were analyzed. A dot indicates the correlation value of an elongated neutrophil. **e** Plot of the correlation of cell length with Mac-1 affinity. Data from two-photon intravital imaging of neutrophils in Mac-1 FRET mice during uropod elongation, as the final step of neutrophil extravasation, were analyzed. A dot indicates the correlation value of an elongated neutrophil

We previously reported that neutrophils are elongated and deposit microparticles at their trailing edge during extravasation and that LFA-1 is important for this process^9^. We also observed that CXCL12 expression in neutrophil-derived microparticles at an early stage of influenza infection is critical for the CD8^+^ T cell-mediated immune response in mice^35^. Thus neutrophil-derived microparticles are important for cell-to-cell interaction during the immune response. In the current study, we investigated the involvement of integrins LFA-1 and Mac-1 in uropod elongation and microparticle formation at the trailing edge of neutrophils during the last step of extravasation in a time-lapse manner. Using two-photon intravital ratiometric CFP/YFP analysis of fMLP-stimulated LFA-1 FRET and Mac-1 FRET mice, we determined the conformational status of LFA-1 and Mac-1 at the trailing edge during uropod elongation. During neutrophil elongation, the leading edge located in the interstitium attempted to move forward; simultaneously, the trailing edge was attached to the endothelial basement membrane. The cell length of an extravasating neutrophil repeatedly became longer until the trailing edge was finally retracted from the endothelial basement membrane. The ratiometric analysis of CFP and YFP signals in LFA-1 FRET and Mac-1 FRET mice indicated that LFA-1 but not Mac-1 adapted a highly active conformation at the trailing edge of elongated neutrophils during extravasation (Fig. 5a, b, Videos 10, 11). Interestingly, although the change in FRET efficiency of LFA-1 was significantly higher than that of Mac-1 (Fig. 5c), neither the conformational status of LFA-1 nor that of Mac-1 in the uropod was specifically related to the elongation length (Fig. 5d, e).

### The duration of perivascular crawling depends on the distance between hotspots I and II in neutrophil extravasation

**Fig. 6:**
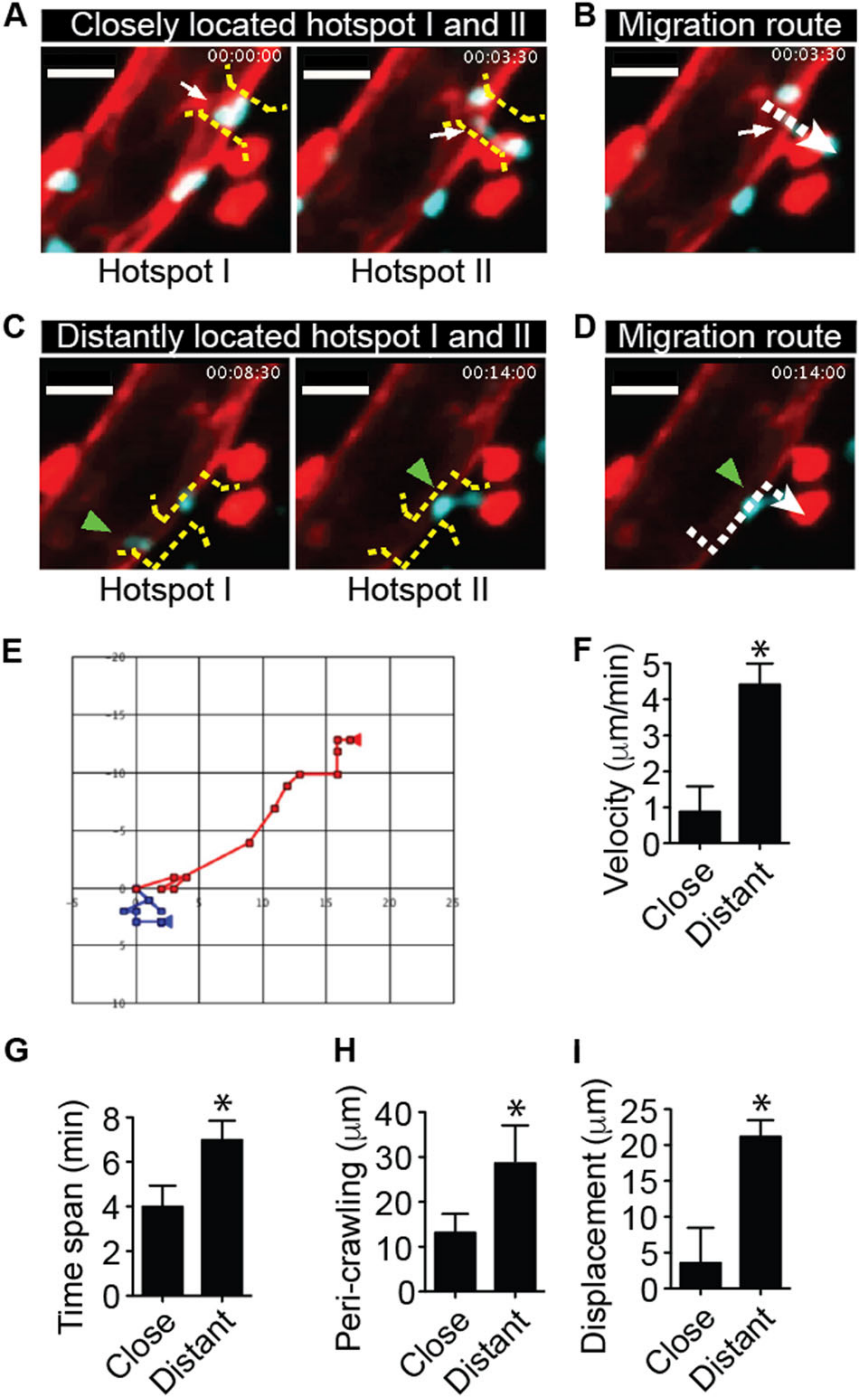
The distance between hotspots I and II determines the perivascular crawling pattern upon stimulation with *N*-formylmethionyl-leucyl-phenylalanine. **a** The neutrophil quickly penetrated the vascular endothelium through closely located hotspots I and II. The white arrow indicates a hotpot, which is located between the yellow dotted brackets. **b** The migration route of the neutrophil through the closely located hotspots I and II is indicated by the white dotted arrow. **c** The neutrophil spent considerable time in the endothelial basement membrane when using the route of distantly located hotspots I and II. The green arrowhead indicates a hotpot between the yellow dotted brackets. **d** The migration route of the neutrophil through the distantly located hotspots I and II is indicated by the green arrowhead. **a**–**d** were generated from Video 12. Scale bar, 20 μm. **e** The migratory patterns of neutrophils using the quick route and round route during extravasation were compared in rectangular coordinates. Unit; μm. Velocity (**f**), time span (**g**), perivascular crawling distance (**h**), and displacement (**i**) were compared between fast penetrating and round penetrating neutrophils. *P < 0.005. A representative dataset from >10 extravasating neutrophils from 3 independent intravital imaging experiments is shown

Perivascular crawling is a step of motility within the endothelial basement membrane^18,36^. In the step of neutrophil perivascular crawling, we found that most of the extravasating neutrophils transmigrated through hotspots I and II. Hotspots I and II could be closely or distantly located at the endothelium. When neutrophils extravasated through the route of closely located hotspots I and II, the cells spent a shorter time and moved slowly compared with neutrophils extravasating through the route of distantly located hotspots I and II (Fig. 6a–d, Video 12). In the case of the close location of hotspots I and II, the neutrophil passed both hotspots I and II quickly, although its velocity was slow. However, when a neutrophil passed through distantly located hotspots I and II, the cell showed frequent turning of direction during perivascular crawling between hotspots I and II, which delays extravasation (Fig. 6e–i). These data suggest that the duration of perivascular crawling depends on whether hotspots I and II are closely or distantly located in the step of neutrophil extravasation. We attempted to quantify the percentages of neutrophils that performed extravasation through closely and distantly located hotspots I and II, respectively. However, it was infeasible to correctly count the numbers of extravasating neutrophils through closely and distantly located hotspots I and II, respectively, only in the beginning stage of inflammation because too many neutrophils were adhered on the endothelium as the inflammation progressed, thus making it impossible to discriminate a neutrophil from adjacently adhered neutrophils.

## Discussion

Structurally, the vascular endothelium consists of the endothelial cell layer, basement membrane, and pericyte sheath. From the viewpoint of leukocyte extravasation and vascular integrity, the vascular endothelium could be considered a double-layered barrier. The vascular endothelial structure forms a barrier that maintains vascular integrity so that blood components circulate only inside the vasculature. In response to tissue damage or infection, leukocytes infiltrate the vascular endothelium to migrate to the inflamed tissue. Among leukocytes, neutrophils are the first major innate immune cell population to penetrate the vascular endothelium. Therefore, understanding the signaling mechanism of the neutrophil extravasation cascade is critical because it is an initial immune response and also affects the motility of other leukocytes. Since the shear stress inside the blood vessels and the vasculature depends on several components, such as the endothelial cell layer, endothelial basement membrane, and pericyte sheath, it is difficult to physiologically mimic the vascular structure in vitro. Therefore, intravital imaging using two-photon microscopy is useful for investigating how neutrophil extravasation in vivo is regulated by adhesion molecules, chemokines, and foreign stimulators. Accordingly, the goals of the current study were to investigate the real-time morphology and motility of extravasating neutrophils in relation to their penetration path through the vascular endothelium. Interestingly, the two-photon intravital microscopic data from the stimulated blood vessels revealed that neutrophils frequently use selective sites for transendothelial migration. We named these sites hotspots I and II. Following the entrance of the endothelial cell layer at hotspot I, the perivascular migrating neutrophils were frequently stuck in the endothelial basement membrane for over 20 min. During this perivascular crawling step, the neutrophils migrated back and forth along the endothelial basement membrane within the pericyte sheath. Since the pericyte sheath forms a type of outer layer of the endothelial basement membrane, the neutrophils subsequently had to find an exit to secede from the endothelial basement membrane for the completion of extravasation. We identified a selective site, hotspot II, for neutrophil exit from the endothelial basement membrane and pericyte sheath. Similar to the entrance site in the endothelial cell layer, hotspot I, the same hotspot II was sequentially used by multiple neutrophils. Therefore, hotspots I and II are conceptually the gates for the entrance and exit of neutrophils, respectively, during their penetration of the vascular endothelium. We suggest that the perivascular crawling of neutrophils is a process of finding an exit from the endothelial basement membrane and pericyte sheath. Thus the duration of perivascular crawling might depend on how quickly a neutrophil finds hotspot II to leave the vascular endothelium. Since hotspots I and II exist adjacently throughout the endothelial cell layer and the endothelial basement membrane, respectively, there would be more than only one route to use a specific set of hotspots I and II. Thus, depending on the distance between hotspots I and II, the duration of the step of perivascular crawling of an extravasating neutrophil is determined. Hotspot II might be the same term for a previously identified site of low laminin expression and pericytes that allows neutrophil infiltration^18^. In summary, the vascular endothelium can act as a physical double-layered barrier that might be minimally disrupted during neutrophil extravasation, which may play a critical role in vascular homeostasis.

We and others have previously reported that LFA-1 and Mac-1 play distinctive roles in leukocyte adhesion and crawling^3,9,37^. Specifically, in the case of neutrophil migration, LFA-1 regulates adhesion to the endothelium, and Mac-1 regulates intravascular crawling^3^. On the other hand, in other leukocytes, such as monocytes and lymphocytes, LFA-1 is involved in crawling^1,37^. Therefore, we attempted to validate how LFA-1 and Mac-1 are distinctly regulated during the neutrophil extravasation cascade. Intravital FRET using two-photon microscopy is useful for visualizing the real-time functionality of target molecules and pathogenesis in living tissues^33,38,39^. To visualize the conformation states of LFA-1 and Mac-1 during the neutrophil extravasation cascade by employing FRET in real time, we generated YFP- and CFP-expressing mice in which the fluorescent proteins were fused with the α and β subunits of these integrins, respectively. These novel mice are the first dual fluorescent knock-in mice to be generated for a real-time functional study of LFA-1 and Mac-1 based on the determination of the CFP-to-YFP ratio. We were able to successfully determine the conformational states of LFA-1 and Mac-1 at the subcellular level in neutrophils. Using two-photon intravital radiometric CFP/YFP analysis of fMLP-stimulated LFA-1 FRET and Mac-1 FRET mice, we intensively investigated the roles of LFA-1 and Mac-1 during the transendothelial migration and uropod elongation stages of neutrophil extravasation. We defined transendothelial migration as entrance in the vascular endothelium, which mainly occurred at hotspot I. At the transendothelial migration step, LFA-1 adapted a higher-affinity conformation than Mac-1. However, Mac-1 clustering was much denser than LFA-1 clustering. Moreover, the expression of Mac-1 was much lower in the leading edge of the transendothelial migratory neutrophils than in the middle and the trailing edge of the neutrophils. Since the leading edge was then located at the basal side of the endothelial cell layer, while the middle and the trailing edge were still in the endothelial cell layer, these data suggest that Mac-1 might be involved in adhesion to the endothelial cell layer. On the other hand, the expression of LFA-1 was uniform in all portions of the neutrophil cell during transendothelial migration. During uropod elongation, i.e., the final step of extravasation, LFA-1 affinity was higher than Mac-1 affinity. Therefore, we propose that the ligand affinity of LFA-1 may be critical at each step of the neutrophil extravasation cascade upon stimulation with fMLP. However, fMLP and IL-8 have been reported to activate and employ Mac-1 and LFA-1 differently during chemotaxis, respectively^13^. Therefore, it might be possible to reveal different patterns of affinity and clustering of LFA-1 and Mac-1 if other stimulators are used to trigger neutrophil migration. LFA-1 FRET and Mac-1 FRET mice are very useful reagents for the investigation of the conformational states of LFA-1 and Mac-1 at the subcellular level during neutrophil extravasation in live animals in a real-time manner.

In the current study, we propose a novel concept: an entrance site, hotspot I, and an exit site, hotspot II, for neutrophil extravasation. The location of hotspot II is closely correlated with the location of the pericyte sheath beneath the endothelial basement membrane because the pericyte sheath is important for neutrophil extravasation^18^. Although transmission electron microscopy clearly visualized the pericyte sheath during neutrophil infiltration of the vascular endothelium, we were able to use two-photon intravital imaging to visualize only the endothelial cell layer by staining blood vessels with an Alexa 594-labeled anti-CD31 antibody or the inside of the blood vessels by intravenous injection of Texas Red/dextran. Therefore, the relationship between hotspot II and the pericyte sheath was not apparent in the current study. However, we previously reported that VLA-3 blocking or inhibition of the VLA-3/laminin interaction by a blocking peptide significantly reduces neutrophil penetration of the endothelial basement membrane^9,40,41^. We also reported that VLA-3 is important for the initial penetration of leukocytes into the endothelial basement membrane. Therefore, VLA-3 might be critical at the exit stage of neutrophils from the endothelium at hotspot II. In summary, whereas LFA-1 and Mac-1 mainly regulate migration within the vascular endothelium, β1 integrins, such as VLA-3, are critical for the forward movement of neutrophils to the interstitium. We previously reported that LFA-1 and VLA-3 collaborate in tiny cell body detachment from the retracting leukocytes during uropod elongation^9^.

Overall, we determined coordinated distinct roles of LFA-1 and Mac-1 during the stages preceding neutrophil protrusion from the endothelial basement membrane and pericyte sheath during neutrophil extravasation, i.e., from the entry of the endothelial cell layer up to exit of the endothelial basement membrane and pericyte sheath.

## Supplementary Information

Supplementary Information

Supplementary Figure 1

Supplementary Figure 2

Video 1

Video 2

Video 3

Video 4

Video 5

Video 6

Video 7

Video 8

Video 9

Video 10

Video 11

Video 12
