# Supplementary material for: LFA-1 (CD11a/CD18) and Mac-1 (CD11b/CD18) distinctly regulate neutrophil extravasation through hotspots I and II
Source: Exp Mol Med. 2019 Apr 9;51(4):39. doi: 10.1038/s12276-019-0227-1 (PMC6456621; doi:10.1038/s12276-019-0227-1)
Supplement: Supplementary file 1 — Supplementary Information [file 12276_2019_227_MOESM1_ESM.docx]

**Supplementary Figures and Videos**

**Figure S1. Generation of LFA-1 and Mac-1 FRET mice.** (A) Gene-targeting procedure for LFA-1 FRET (CD18-mCFP/CD11a-mYFP) knock-in mice and Mac-1 FRET (CD18-mCFP/CD11b-mYFP) knock-in mice. The mCFP sequence was knocked into the C-terminus of the mouse integrin CD18 subunit. The mYFP sequence was knocked into the C-terminus of the mouse integrin CD11a or CD11b subunit. (B). Schematic representation of LFA-1 and Mac-1 activation. In the basal, bent conformation, the cytoplasmic domains of the α and β subunits of LFA-1 and Mac-1 are in close proximity. In this case, energy transfer occurs between CFP (CD18-mCFP) and YFP (CD11a-mYFP or CD11b-mYFP). In the active, extended conformation, there is low or no FRET because of the distal location of the cytoplasmic domains. Ex, extracellular domain; TM, transmembrane; and Cy, cytoplasmic domain. (C) Mating of CD18-mCFP knock-in mice with CD11a-mYFP and CD11b-mYFP knock-in mice generated LFA-1 FRET (CD18-mCFP/CD11a-mYFP) mice and Mac-1 FRET (CD18-mCFP/CD11b-mYFP) mice, respectively. (D) CFP and YFP expression in bone marrow cells isolated from LFA-1 FRET mice and Mac-1 FRET mice was visualized. Scale bars, 10 μm. (E) Western blotting analyses of CFP and YFP were performed with a polyclonal anti-GFP antibody in bone marrow cells from WT, LFA-1 FRET, and Mac-1 FRET mice (left panel). The lower and upper bands indicate CFP-conjugated CD18 and YFP-conjugated CD11a or CD11b. As expected, no band was detected in WT mice. For the western blot analysis of the CD18 integrin, bone marrow cells from WT, LFA-1 FRET, and Mac-1 FRET mice were used. The molecular mass of CD18 molecules from LFA-1 FRET mice and Mac-1 FRET mice was higher than that from WT CD18, consistent with the molecular mass of CFP. A representative image from experiments repeated three times is shown. (F) CD11a, CD11, and CD18 expression in bone marrow cells were analyzed to compare the cell surface expression of these integrins in WT, LFA-1 FRET, and Mac-1 FRET mice. MFI, mean fluorescence intensity.

**Figure S2. Optimization of the wavelength for two-photon microscopy for CFP and YFP visualization in LFA-1 FRET mice and Mac-1 FRET mice.** A wavelength scan was performed to compare the intensities of CFP and YFP in neutrophils from LFA-1 FRET mice and Mac-1 FRET mice. At 840 nm, the fluorescence intensities of CFP and YFP were similar, resulting in an approximate value of 1 for the CFP to YFP ratio.

**Video 1. Neutrophil infiltration in LysM-GFP mice upon stimulation with fMLP.**

**Video 2. Continuous infiltration of neutrophils through the hotspot in LysM-GFP mice upon stimulation with fMLP.**

**Video 3. Substeps of neutrophil extravasation in LysM-GFP mice upon stimulation with fMLP.**

**Video 4. Three-dimensional image of a neutrophil during transendothelial migration in LysM-GFP mice upon stimulation with fMLP.**

**Video 5. Embedment of neutrophils in the endothelial basement membrane during extravasation in LysM-GFP mice upon stimulation with fMLP (1).**

**Video 6. Embedment of neutrophils in the endothelial basement membrane during extravasation in LysM-GFP mice upon stimulation with fMLP (2).**

**Video 7. Continuous penetration of the endothelium through the same spot during extravasation in LysM-GFP mice upon stimulation with fMLP.**

**Video 8. Intensities of CD11a-mYFP and CD18-mCFP during transendothelial migration in CD11a-mYFP/CD18-mCFP mice upon stimulation with fMLP.**

**Video 9. Intensities of CD11b-mYFP and CD18-mCFP during transendothelial migration in CD11b-YFP/CD18-mCFP mice upon stimulation with fMLP.**

**Video 10. Intensities of CD11a-mYFP and CD18-mCFP as well as the ratio of CFP/YFP during elongation in CD11a-mYFP/CD18-mCFP mice upon stimulation with fMLP.**

**Video 11. Intensities of CD11b-mYFP and CD18-mCFP as well as the ratio of CFP/YFP during elongation in CD11b-mYFP/CD18-mCFP mice upon stimulation with fMLP.**

**Video 12. Close and distant routes of hotspots I and II during neutrophil extravasation in CD18-mCFP mice upon stimulation with fMLP.**
