# Supplementary figures and images for: LFA-1 (CD11a/CD18) and Mac-1 (CD11b/CD18) distinctly regulate neutrophil extravasation through hotspots I and II
Source: Exp Mol Med. 2019 Apr 9;51(4):39. doi: 10.1038/s12276-019-0227-1 (PMC6456621; doi:10.1038/s12276-019-0227-1)

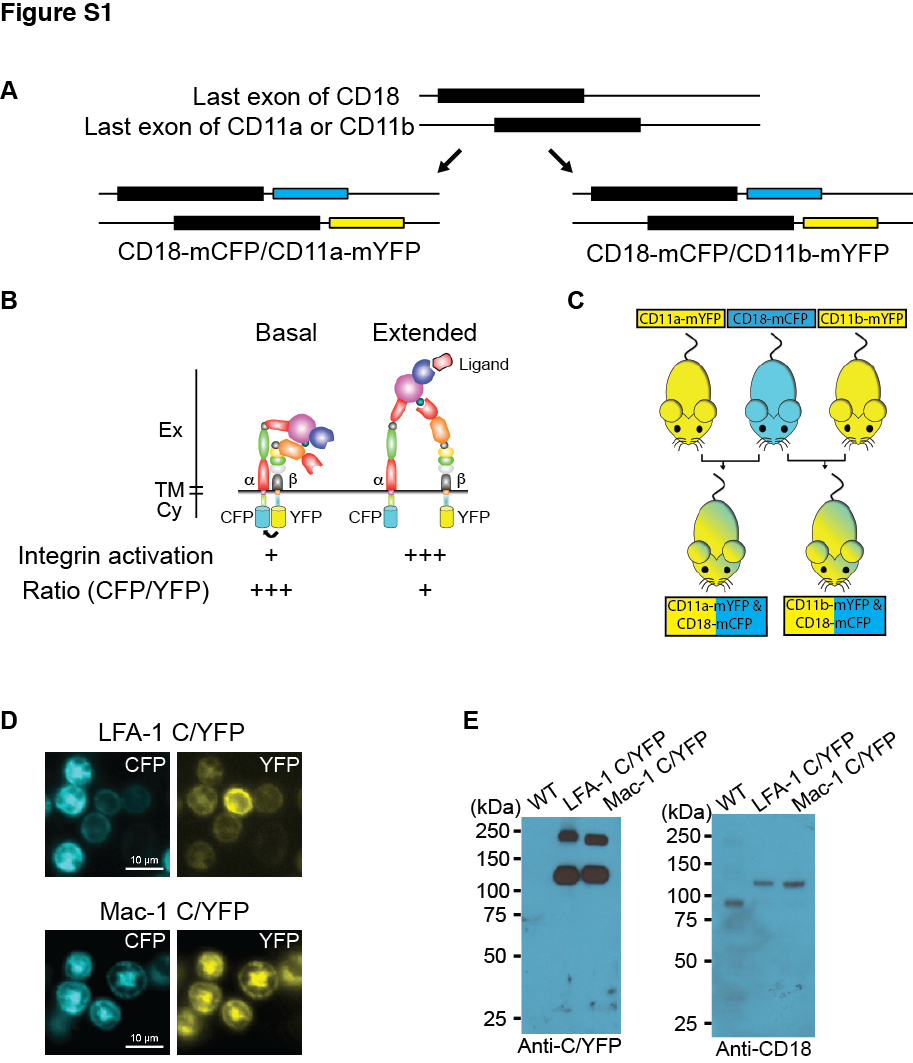

Supplement: Supplementary file 2 — Supplementary Figure 1 [file 12276_2019_227_MOESM2_ESM.png]

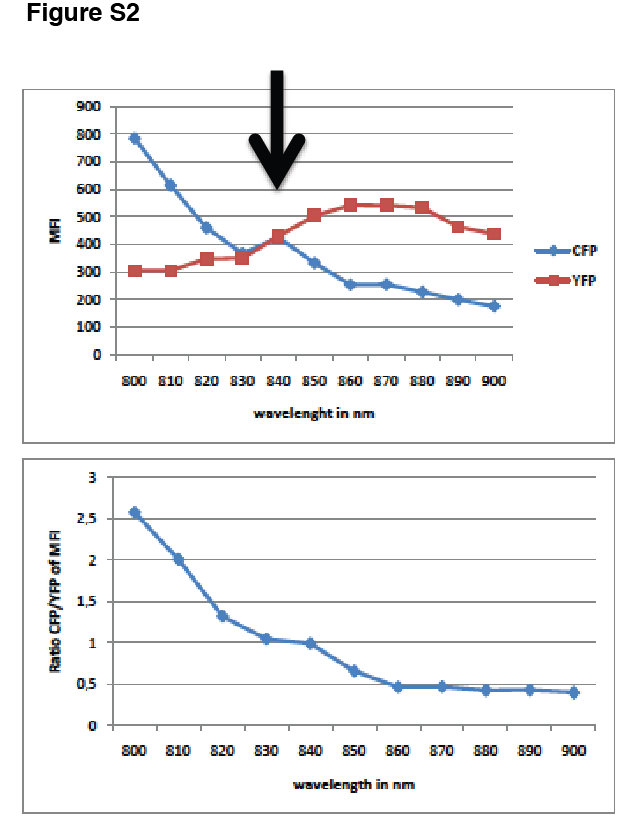

Supplement: Supplementary file 3 — Supplementary Figure 2 [file 12276_2019_227_MOESM3_ESM.png]
